# Increasing the public activity cliff knowledge base with new categories of activity cliffs

**DOI:** 10.2144/fsoa-2020-0020

**Published:** 2020-04-15

**Authors:** Huabin Hu, Jürgen Bajorath

**Affiliations:** 1 Department of Life Science Informatics, B-IT, LIMES Program Unit Chemical Biology & Medicinal Chemistry, Rheinische Friedrich-Wilhelms-Universität, Endenicher Allee 19c, Bonn D-53113, Germany

**Keywords:** active compounds, activity cliffs, computational analysis, medicinal chemistry, open access data, privileged substructures, structural isomers, substitution sites

## Abstract

**Aim:**

Extending the public knowledge base of activity cliffs (ACs) with new categories of ACs having special structural characteristics.

**Methodology:**

Dual-site ACs, isomer ACs and ACs with privileged substructures are described and their systematic identification is detailed.

**Exemplary results & data:**

More than 7400 new ACs belonging to different categories with activity against more than 200 targets were identified and are made publicly available.

**Limitations & next steps:**

For dual-site ACs, limited numbers of isomers are available as structural analogs for rationalizing contributions to AC formation. The search for such analogs will continue. In addition, the target distribution of ACs containing privileged substructures will be further analyzed.

## Lay abstract

Activity cliffs (ACs) are formed by small molecules that have very similar structures, are active against the same biological target, but have a large difference in potency against their target. Accordingly, ACs are of interest in medicinal chemistry because they reveal small structural changes that greatly influence the potency of active compounds. This information can be used for compound optimization. Computational methods are applied to search for ACs in large compound databases. Here, we further extend the public AC knowledge base with new categories of ACs having special structural characteristics.

## Graphical abstract

Shown are an exemplary dual-site activity cliff (AC) (top), isomer AC (middle) and an AC containing a privileged substructure (bottom). Structural differences between AC compounds are highlighted in blue and the privileged substructure is colored pink. Compound targets and potency (pK_i_) values are reported.

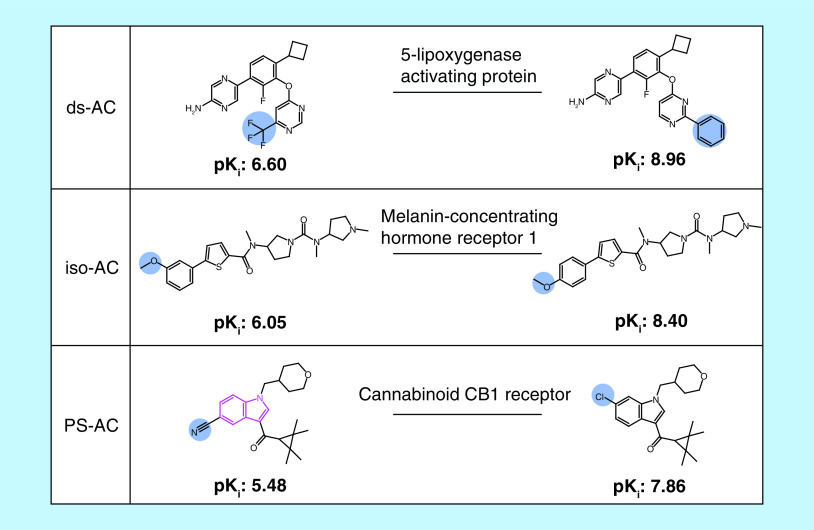

Activity cliffs (ACs) are defined as pairs or groups of structurally similar (analogous) compounds that are active against the same target but have a large difference in potency [1–4]. ACs have also been studied on the basis of compounds, which are highly potent against a given target and structural analogs that are confirmed inactive against this target [4]. Furthermore, ACs have been investigated from a variety of research perspectives including the consideration of different AC concepts, different types of data analysis and AC predictions [1–9]. In medicinal chemistry, ACs are of particular interest because they capture small chemical modifications of active compounds that substantially contribute to, or determine, structure–activity relationships (SARs) [2,3].

For formally defining ACs, molecular similarity and potency difference criteria must be specified [2–4]. Similarity can be calculated on the basis of chemical descriptors and numerical similarity metrics (descriptor-based/numerical similarity) or on the basis of substructure relationships (substructure-based similarity) [3,4]. Substructure-based similarity measures include shared scaffolds, the formation of matched molecular pairs (MMPs) or membership in the same analog series (AS) [4,10,11]. Compounds forming MMP-based ACs are confined to chemical changes at a single substitution site [10,12], whereas AS-based ACs may contain single or multiple substitution sites [11,13].

Furthermore, for defining ACs, constant potency difference thresholds can be applied across different compound activity classes (e.g., at least 100-fold potency difference) [2,3]. Alternatively, activity class-dependent potency difference thresholds can be determined on the basis of statistically significant potency differences, with respect to intra-class potency value distributions [14,15]. In either case, the use of assay-independent equilibrium constants (K_i_ values) as potency measurements is generally preferred over assay-dependent measurements such as IC_50_ values. The use of K_i_ values makes it possible to compare ACs for a given target and across different targets in a meaningful way.

Considering the evolution of the AC concept in medicinal chemistry [4], we have defined three generations of ACs [4,16], depending on the structural similarity and potency difference criteria that are applied:

## *First generation* ACs

Similarity criterion: numerical or substructure-based similarity.

Potency difference criterion: constant potency difference threshold across all activity classes.

## *Second generation* ACs

Similarity criterion: MMP formation (analog pairs with single substitution site).

Potency difference criterion: variable activity class-dependent potency difference thresholds.

## *Third generation* ACs

Similarity criterion: structural analogs originating from the same AS (with single or multiple substitution sites).

Potency difference criterion: variable activity class-dependent potency difference thresholds.

Previously, we have generated a large collection of second generation ACs [15,17] and made it publicly available as an open access deposition [17,18].

For nearly 100 different activity classes, each representing a unique target protein, more than 20,000 activity class-dependent ACs were identified, also taking structural analogs of potent compounds into account that were confirmed inactive against the same target [15,17]. Compound activity data were extracted from medicinal chemistry sources (ChEMBL database) [19] and high-throughput screens (PubChem Bioassays) [20].

Herein, we further increase the public AC knowledge base through addition of three recently introduced categories of ACs including dual-site ACs (ds-ACs) [13], isomer ACs (iso-ACs) [21] and ACs containing ‘privileged substructures’ (PS-ACs) [22]. These AC categories are detailed in the methodology section and an in-depth analysis of each category is reported in its original publication.

The PS concept was originally introduced in medicinal chemistry by Evans *et al.* [23] and has become increasingly popular over time [24,25]. PSs are frequently found in compounds with preferential activity against specific target families. They usually are not selective for a particular target but display a tendency of preferential binding to an individual target family over others. Accordingly, PSs are often considered as family-directed core structures in medicinal chemistry [23–25]. Studying PS-ACs is attractive because these ACs reveal different levels of SAR information associated with individual PSs, as described in detail [22].

In the following, we report a systematic search for ds-ACs, iso-ACs and PS-ACs, resulting in a new collection of ACs that further extends our knowledge base of ACs and is made available as an open access deposition. Importantly, ds-ACs, iso-ACs and PS-ACs were originally introduced in independent studies. Herein, we report a new unified search strategy that has made it possible to identify these ACs in bioactive compounds in concert applying consistent criteria, determine the overlap between different AC categories and study ACs belonging to these categories. This strategy is related to, yet distinct from the one applied in the original assessment of PS-ACs, which were most recently introduced [22], and has yielded the first public collection of PS-ACs. All new ACs identified in our systematic analysis are made freely available as a part of this study, providing a wealth of examples for follow-up investigations.

## Methodology

### Compound activity data

Bioactive compounds were extracted from the latest version of the ChEMBL [19] database (release 25). For selection of high-confidence activity data, rigorous criteria were applied. Only compounds forming direct interactions with human targets (i.e., assay relationship type ‘D’) at the highest assay confidence level (i.e., assay confidence score 9) were selected. Furthermore, only equilibrium constants (i.e., K_i_ values) with specified numerical values (‘=’ relationship) were accepted as potency measurements for given targets.

### Privileged substructures

**Figure 1.**
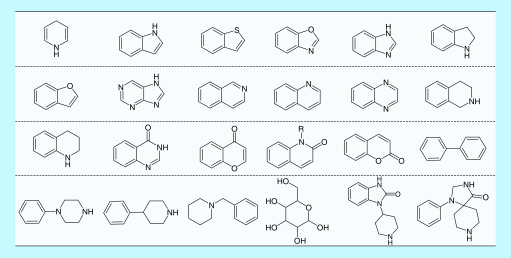
Privileged substructures. Shown are the structures of 24 privileged substructures found in at least 100 unique bioactive compounds.

PSs were defined according to Welsch *et al.* [25]. A systematic search was carried out for PSs that were contained in 100 or more unique ChEMBL compounds. Figure 1 shows 24 PSs that were identified and further considered for AC analysis.

### Compound fragmentation

To ensure consistent generation of ACs belonging to different categories, a recently introduced compound fragmentation scheme was applied [22]. Using a decomposition algorithm for MMP generation [10], exocyclic single bonds in compounds were systematically fragmented, yielding two fragments per step. During the fragmentation process, the following size restrictions were applied to obtain a core and substituent fragment [12]. The number of nonhydrogen atoms of the core fragment was required to be at least twice as large as the number of nonhydrogen atoms comprising the substituent fragment. In addition, the size of the substituent was restricted to at most 13 nonhydrogen atoms. Furthermore, the substituent in the core fragment was replaced by a hydrogen atom (R → H). The calculations were carried out using in-house scripts with the aid of the OpenEye chemistry toolkit (NM, USA) (version 1.7.7) [26].

### Analog pairs & activity cliffs

Following fragmentation, compounds having the same activity and sharing the same core were organized into individual sets of analogs. Then, analog pairs (APs) differing at two substitution sites were systematically enumerated and categorized as follows:

- Structural isomers: the same substituent occurred at two different sites (core positions).
- Dual-site analogs: two different substituents occurred at different sites. The size difference between these exchanged substituents was restricted to at most eight non-hydrogen atoms.

**Figure 2.**
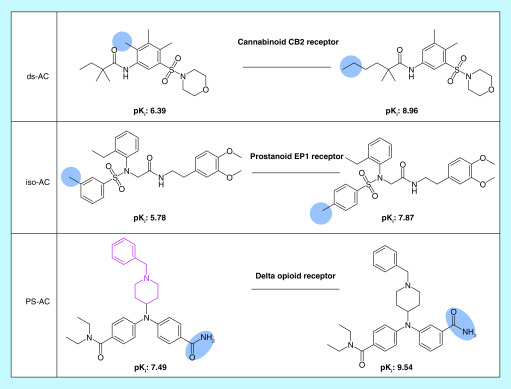
Exemplary activity cliffs belonging to different categories. From the top to the bottom, an exemplary ds-AC, iso-AC and PS-AC are shown. For each AC, the target and compound potency (pK_i_) values are reported. Structural modifications and the PS are colored blue and pink, respectively. AC: Activity cliff; ds-AC: Dual-site activity cliff; iso-AC: Isomer activity cliff; PS: Privileged substructure.

For each AP, it was determined whether the participating compounds had an at least 100-fold difference in potency, which qualified the pair as an iso-AC or ds-AC. We note that iso-ACs are confined to structural isomers and hence distinct from chirality or chiral cliffs [4,9] where cliff compounds are distinguished by different chirality at a given stereocenter. Furthermore, for each AC, it was determined if it contained a PS. ACs with PSs were also classified as PS-ACs. Figure 2 shows exemplary ACs belonging to different categories. By definition, iso-ACs represent a special case of ds-ACs.

### Detection of isomers of dual-site activity cliff compounds

For ds-AC compounds, a further systematic search for structural isomers (from the same activity class) with the same substituent at the other substitution site was carried out. If such isomers were identified, it was possible to generate an extended data structure for a ds-AC, revealing the contributions of substituent positions to AC formation, as further discussed below.

## Exemplary results & data

### Unified search strategy for activity cliffs belonging to different categories

**Figure 3.**
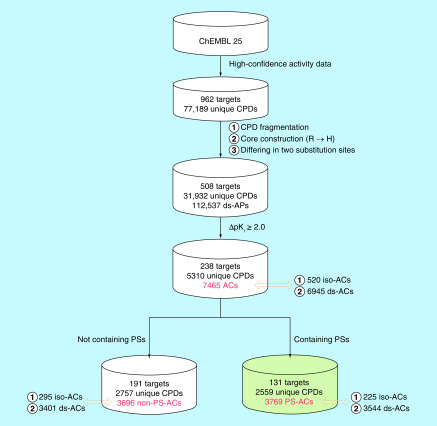
Unified search strategy for the identification of different activity cliffs. The identification of ds-ACs, iso-ACs and PS-ACs is summarized. Numbers of compounds, targets and ACs are given at each stage. AC: Activity cliff; CPD: Compound; ds-AC: Dual-site activity cliff; ds-AP: Dual-site analog pair; iso-AC: Isomer activity cliff; PS: Privileged substructure; PS-AC: Privileged substructure-containing activity cliff.

Originally, ds-ACs, iso-ACs and PS-ACs were separately studied. Here, we have implemented a unified search strategy to identify ACs belonging to these categories in parallel and determine their overlap. The search strategy is summarized in Figure 3. After compound fragmentation, a total of 112,537 qualifying APs were identified that yielded a total of 7465 ACs, which were assigned to different categories, as further detailed below.

### Extended dual-site activity cliff data structure

**Figure 4.**
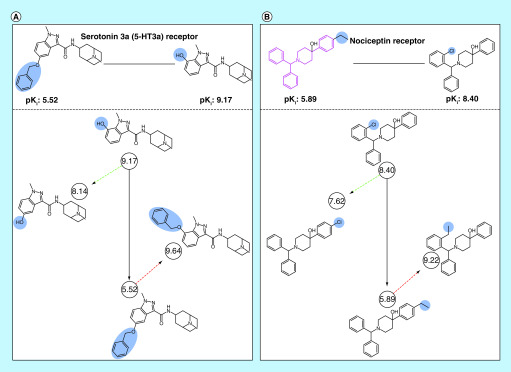
Extended data structure for dual-site activity cliffs. Shown are exemplary ds-ACs and corresponding data structures **(A)** without and **(B)** with a PS. Highly and weakly potent ds-AC compounds are connected by solid black arrows. Structural isomers of highly and weakly potent AC compounds are connected to corresponding ds-AC compounds using dashed green and red arrows. Structural modifications and the PS are colored blue and pink, respectively. For each example, the target name and compound potency (pK_i_) values (in circles) are reported. AC: Activity cliff; ds-AC: Dual-site activity cliff; PS: Privileged substructure.

For SAR exploration, ds-ACs can be extended to generate a data structure comprising four analogs, as illustrated in Figure 4. This data structure makes it possible to examine the contributions of substituent positions to ds-AC formation and is thus rich in SAR information [13]. Its generation requires the identification of isomers of ds-AC compounds with inversely repositioned substituents, as shown in Figure 4. A systematic search for such isomers revealed that 396 ds-ACs could be fully extended with two qualifying isomers. In addition, for 2320 other ds-ACs, only one isomer was identified. Among analogs from different series, structural isomers are statistically under-represented when compared with analogs carrying different substituents. A possible reason might be that medicinal chemists, from an SAR perspective, typically prefer introducing different substituents at a given site, rather than synthesizing analogs with a ‘moving’ substituent (structural isomers). Regardless, the extended data structure based upon ds-ACs offers additional opportunities for SAR analysis and illustrates the utility of this AC category.

### Data

Our systematic search identified a total of 3696 ACs without PSs that were formed by 2757 unique compounds with activity against 191 targets. These ACs included 3401 ds- and 295 iso-ACs. Thus, only a limited number of iso-ACs were available. In addition, the search identified 3769 PS-ACs formed by 2559 unique compounds with activity against 131 targets. These PS-ACs included 3544 ds- and 225 iso-ACs. ACs with and without PSs shared 84 targets.

Our analysis revealed that approximately half of the newly identified ACs contained one of 24 predefined PSs that were detected in at least 100 unique bioactive compounds. The high frequency with which a predefined set of PSs occurred in ds- and iso-ACs, thus combining different AC categories, indicated that PSs yielded SAR-informative compounds with potential for further optimization. Hence, on the basis of AC analysis, these PSs deserve further consideration in medicinal chemistry. The PS-ACs provided as a part of our study should aid in further exploring these PSs.

### Data deposition

All ACs identified herein are provided in a text file. For each AC, category membership(s) are specified. For AC compounds, the ChEMBL ID, canonical SMILES representation and potency value are reported. For PS-ACs identified herein (forming a subset of iso-ACs and ds-ACs), the SMILES string of the PS is also provided. The data are made freely available as a deposition on the ZENODO open access platform [27].

## Limitations & next steps

The extended ds-AC data structure enables the analysis of substitution site-specific contributions to AC formation. However, among structural analogs, structural isomers are under-represented and only limited numbers of isomers are currently available for ds-AC analysis. This is essentially the only data-dependent limitation associated with exploring the new AC categories introduced here. Hence, the search for isomers as structural analogs for ds-AC analysis will continue. Furthermore, the large number of PS-ACs we identified makes it possible to investigate the target distribution and SAR information content of PS-containing compounds and their analogs in greater detail. For this purpose, PS-ACs provide immediate focal points.

**Executive summary**

- The activity cliffs (AC) concept is rationalized.
- Different generations of ACs are defined.

**Methodology**

- Procedures for AC identification are detailed.
- Recently introduced AC categories are described.
- Search routines are implemented.

**Exemplary results & data**

- A unified search strategy for identifying different ACs is detailed.
- Search results are summarized.
- An extended data structure based upon dual-site ACs is introduced.
- A collection of ACs is generated.
- Details of its open access deposition are provided.

**Limitations & next steps**

- Limited availability of isomers of dual-site AC compounds is discussed.
- Further analysis of privileged substructure-containing ACs is proposed.
